# RACIAL/ETHNIC DISPARITIES IN ALZHEIMER’S DISEASE RISK: ROLE OF EXPOSURE TO AMBIENT FINE PARTICLES

**DOI:** 10.1093/geroni/igad104.0717

**Published:** 2023-12-21

**Authors:** Mark Espeland, Diana Younan, Xinhui Wang, Tara Gruenewald, Margaret Gatz, Marc Serre, William Vizuere, Jiu-Chiuan Chen

**Affiliations:** Wake Forest University School of Medicine, Winston-Salem, North Carolina, United States; University of Southern California, Los Angeles, California, United States; University of Southern California, Los Angeles, California, United States; Chapman University, Orange, California, United States; University of Southern California, Los Angeles, California, United States; University of North Carolina - Chapel Hill, Chapel Hill, North Carolina, United States; University of North Carolina - Chapel Hill, Chapel Hill, North Carolina, United States; University of Southern California, Los Angeles, California, United States

## Abstract

Whether racial/ethnic disparities in Alzheimer’s disease (AD) risk may be explained by ambient fine particles (PM2.5) has not been studied. We conducted a prospective, population-based study on a cohort of Black (n=481) and White (n=6004) older women (aged 65– 79) without dementia at enrollment (1995–98). Cox models accounting for competing risk were used to estimate the hazard ratio (HR) for racial/ethnic disparities in AD (1996–2010) and the association with time-varying annual average PM2.5 (1999–2010) estimated by spatiotemporal model. Over an average follow-up of 8.3 (±3.5) years with 158 incident cases (21 in Black women), the racial disparities in AD risk (range of adjusted HRBlack women=1.85–2.41) observed in various models could not be explained by geographic region, age, socioeconomic characteristics, lifestyle factors, cardiovascular risk factors, and hormone therapy assignment. Estimated PM2.5 exposure was higher in Black (14.38±2.21 µg/m3) than in White (12.55±2.76 µg/m3) women, and further adjustment for the association between PM2.5 and AD (adjusted HRPM2.5=1.18–1.28) slightly reduced the racial disparities by 2%–6% (HRBlack women=1.81–2.26). The observed association between PM2.5 and AD risk was ~2 times greater in Black (HRPM2.5=2.10–2.60) than in White (HRPM2.5=1.07–1.15) women (range of interaction Ps: <.01–.01). We found similar results after further adjusting for social engagement (social strain, social support, social activity, living alone), stressful life events, Women’s Health Initiative’s clinic sites, and neighborhood socioeconomic characteristics. PM2.5 may contribute to racial/ethnic disparities in AD risk and its associated increase in AD risk was stronger among Black women.

